# Radiographic Features of Metastatic Brain Tumors from ALK-rearranged Non-small Cell Lung Cancer: Implications for Optimal Treatment Modalities

**DOI:** 10.7150/jca.30091

**Published:** 2019-10-22

**Authors:** Li Chu, Jianjiao Ni, Xi Yang, Tong Tong, Jialei Wang, Fang Yin, Ruimin Li, Yida Li, Liqing Zou, Yuan Li, Congying Xie, Guodong Li, Zhengfei Zhu

**Affiliations:** 1 Department of Radiation Oncology, Fudan University Shanghai Cancer Center, Shanghai, China; 2 Department of Radiology, Fudan University Shanghai Cancer Center, Shanghai, China; 3 Department of Medical Oncology, Fudan University Shanghai Cancer Center, Shanghai, China; 4 Center for Drug Clinical Research, Shanghai University of Traditional Chinese Medicine, Shanghai 201203, China; 5 Department of Pathology, Fudan University Shanghai Cancer Center, Shanghai, China; 6 Radiotherapy and Chemotherapy Department, the 1st Affiliated Hospital of Wenzhou Medical University, Wenzhou, China; 7 Department of Interventional Radiology, Fudan University Shanghai Cancer Center, Shanghai, China; 8 Department of Oncology, Shanghai Medical College, Fudan University, Shanghai 200032, China

**Keywords:** lung cancer, ALK, brain metastases, radiographic feature

## Abstract

**Purpose:** To investigate the radiological features on magnetic resonance imaging (MRI) of brain metastases (BM) from* ALK*-rearranged non-small cell lung cancer (NSCLC).

**Patients and Methods:** We retrospectively evaluated data from 40 eligible patients with *ALK*-rearranged NSCLC. Radiographic features of metastatic brain tumors, including the number, size, location, and peritumoral brain edema size (PBES), were delineated using MRI.

**Results:** 13 patients had metachronous BM (MBM), having developed BM at least 6 months after diagnosis with NSCLC. The remaining patients were categorized as having synchronous BM (SBM). Compared with patients in the SBM group, patients in the MBM group were found to have more favorable values for radiological features including BM number, BM size, and PBES. Ten (76.9%) of the 13 patients with MBM had ≤3 lesions and were asymptomatic, and none had developed a diffuse BM pattern, supporting the adoption of stereotactic radiosurgery (SRS) in the majority of these patients and against the administration of prophylactic cranial irradiation (PCI). Conversely, among the 27 patients with SBM, 15 (55.6%) patients had >3 lesions and 12 (44.4%) patients were symptomatic, highlighting the necessity of rapidly administrating brain radiotherapy, either as SRS or whole brain radiotherapy (WBRT). Importantly, only two patients (5.0%) had metastases in the hippocampus and peri-hippocampus region, and both were in the SBM group, indicating the feasibility of hippocampal avoidance WBRT in *ALK*-rearranged NSCLC.

**Conclusions:** Both WBRT and SRS are appropriate for the treatment of BM in patients with *ALK*-rearranged NSCLC. The incidence of BM in the hippocampus and peri-hippocampus region is low in our radiological data. Nearly 80% of patients with metachronous BM have oligo-metastatic lesions, indicating that SRS is the preferred therapy while PCI is not indicated.

## 1. Introduction

Lung cancer is a common malignancy, meaning that the identification of even minor subsets of lung cancer patients on the basis of specific genetic alterations can translate into large cohorts. Anaplastic lymphoma kinase (*ALK*) gene rearrangements are detected in 3%-7% of patients with lung adenocarcinoma 1-2. Furthermore, *ALK*-rearranged non-small cell lung cancer (NSCLC) is associated with a 50% lifetime incidence of brain metastases (BM), and several studies have shown that patients with NSCLC BM harboring *ALK* rearrangements have prolonged survival compared with other NSCLC subgroups, with median survival exceeding 4 years and a 2-year survival rate of approximately 70% 3-4.

Considering the high incidence of BM and the relatively long survival of patients with *ALK*-positive NSCLC, the optimal treatment for this subgroup should balance therapeutic effects and treatment toxicities. Thus, we conducted the present study to explore the radiographic features and clinical characteristics of *ALK*-positive NSCLC patients with BM. It is anticipated that the resulting data will inform the selection of appropriate treatment modalities, especially radiotherapy, for this patient population.

## 2. Patients and Materials

### 2.1. Patients

Data from all patients treated for lung cancer patients at Fudan University Shanghai Cancer Center between March 2008 and May 2017 were initially screened from the database of the Department of Pathology. The inclusion criteria were: 1) patients with pathologically confirmed NSCLC; 2) *ALK* rearrangements verified both by fluorescence *in situ* hybridization (FISH) and Ventana immunohistochemistry (IHC); and 3) BM reviewed and confirmed at our center by two experienced radiologists based on gadolinium (Gd)-enhanced magnetic resonance imaging (MRI). We selected patients irrespective of the timing of BM occurrence; thus, patients were classified as synchronous BM (SBM) and metachronous BM (MBM) according to the time interval to development of BM. Patients with SBM had brain metastases diagnosed within 6 months of NSCLC diagnosis, while those with MBM had no BM at initial diagnosis but developed BM after >6 months. Clinical data including time of cancer diagnosis, age at diagnosis, sex, smoking history, and neurological symptoms were also collected from the electronic records.

### 2.2. Molecular analysis for *ALK* rearrangements

At our center, *ALK* rearrangements are routinely detected using both FISH and Ventana IHC. ALK IHC was performed using a Ventana BenchMark XT automated slide-processing system (Ventana Medical Systems, Basel, Switzerland) according to the manufacturer's protocol, and using anti-ALK primary antibody (D5F3, Ventana Medical Systems), an Opti-View DAB immunohistochemistry detection kit (Ventana Medical Systems), and an OptiView amplification kit (Ventana Medical Systems). For IHC analysis, strong granular cytoplasmic staining in tumor cells was classified as positive, and the absence of strong granular cytoplasmic staining was classified as negative. Moreover, our Department of Pathology further confirmed *ALK* gene rearrangements by FISH, and identified that the percentage of nuclei with break-apart or isolated red signals was 32% 5-6.

### 2.3. Radiographic features of BM

**Figure 1:**
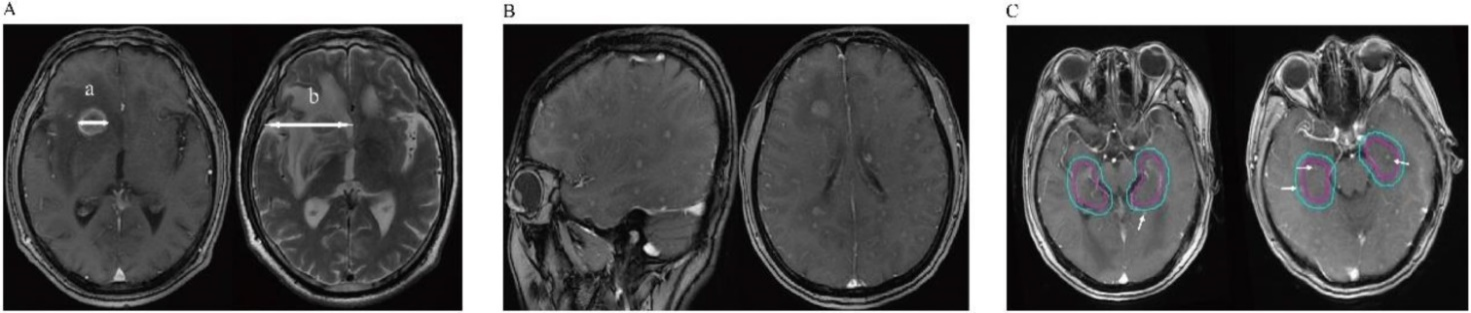
** A:** Detailed method of evaluating metastatic brain tumors; The maximum diameter (a) of the tumor (TS) was measured in T1-weighted Gd-enhanced image. Then we also measured the maximum diameter of its associated peritumoral brain edema (PTBE) (b) at the same slice of the T2-weighted image. The “peritumoral brain edema index” (PBEI) was defined as (b-a)/a. **B:** T1-weighted Gd-enhanced image demonstrates the patient with diffuse brain metastases. **C:** Two patients with hippocampus and Peri-Hippocampus region metastases in SBM group. The red circle means the hippocampus area, the blue circle means the Peri-Hippocampus region.

MRI is routinely used at our center to diagnose BM. All scanning was performed using the same 1.5 T MRI machine (GE Healthcare, Waukesha, WI). MRI studies included pre- and post-Gd administration sequences. The images obtained before administering contrast agent were axial T1- and T2-weighted images (6-mm sections, 1-mm gap), and sagittal T1-weighted images (4-mm sections, contiguous). The images obtained after administering contrast agent were axial and coronal thin-section images (4-mm, contiguous), and T1-weighted images (fast spoiled gradient-recalled echo acquisition [FSPGR]; repetition time: 120-215; echo time: 2.2-2.8). For each patient, brain MRI scans were reviewed by two experienced radiologists, and three variables reflecting radiological features were measured: 1) BM number; 2) BM size on T1-weighted Gd-enhanced imaging; and 3) peritumoral brain edema size (PBES) on T2-weighted imaging. For patients with multiple brain tumors, the largest lesion was accurately measured in one dimension (the longest diameter along the plane of measurement), and this value was used for analysis 7. PBES was calculated by subtracting the diameter measured on T1-weighted Gd-enhanced imaging from the diameter on T2. If no peritumoral brain edema (PBE) was observed, the edema size was evaluated as zero. As shown in Figure 1A and reported previously, PBES was found to be vasogenic edema, and was associated with tumor size 8-10. Therefore, we subsequently refer to the “vasogenic edema ratio” as previously reported by Tung *et al*. 11. To minimize the effect of tumor size on PBES, we defined the relationship between the tumor and its associated PBES as the peritumoral brain edema index (PBEI), calculated by dividing PBES by tumor size. Thus, for PBES and PBEI, the larger the value, the greater the degree of PBE present. Hippocampal or peri-hippocampal metastases were defined as metastatic lesions in the region of the hippocampus and within a 5-mm margin (hippocampus and peri-hippocampus (PH) region), evaluated by enhanced MRI as described previously for the RTOG 0933 trial 12.

### 2.4. Statistical analysis

Continuous variables were summarized using descriptive statistics, such as means and standard deviations (SD) or medians and ranges. Categorical variables were tabulated by frequency and percentage. Fisher's exact test was used to compare sex, smoking history, pathology and initial neurological symptoms. The t test was used to compare age. Kaplan-Meier estimates of median Overall Survival (OS) with 95% confidence intervals (CIs). The numbers of BM between two groups were analyzed using a two-sided Fisher's exact test. The Wilcoxon rank sum test was applied to comparisons of the size of the largest brain lesion, PBES, and PBEI. *P*-values <0.05 were considered statistically significant. All analyses were performed using SAS version 9.3 (SAS Institute, Cary, NC, USA).

## 3. Results

A total of 40 patients were selected according to the inclusion criteria, including 13 (32.5%) and 27 (67.5%) in the MBM and SBM groups, respectively. Clinical characteristics of the patients are shown in Table 1. Briefly, the majority of patients had adenocarcinomas, the median age was 50 years (range: 22-66 years), and most patients were never smokers, which was consistent with the general characteristics of patients with *ALK*-positive NSCLC 13. All 13 patients with MBM patients had been treated with platinum-based chemotherapy before the occurrence of brain metastases, while only 3 patients had received crizotinib. OS (according to the diagnosis time to death) was 65 months (95%CI: 10.7-119.4, range 13-147) versus not reached (*p*=0.7727) in the MBM and SBM groups. There was no significant difference in clinical characteristics between the two groups, although more patients in the SBM group experienced neurological symptoms compared with the MBM group. In our center, most patients with NSCLC harboring *ALK* rearrangements underwent regular brain MRI follow-up at intervals of 2-3 months, even in the absence of baseline BM.

The radiological features of patients are summarized in Table 2. In total, 13 and 27 BM patients in the MBM and SBM groups, respectively. A higher proportion of patients in the MBM group had ≤3 BM. 3 patients had >3 BM (8, 4, 4), the lesions were stable or decreasing after chemotherapy (2 patients) or target therapy (1 patient) without local BM treatment, meaning that the patients were more suitable for stereotactic radiation therapy according to current guidelines 14. Only one patient (in the SBM group) showed a diffuse BM pattern as described elsewhere (Figure 1B). Additionally, we found that patients in the MBM group had more favorable results for radiological features including BM size, PBES, and PBEI than those in the SBM group, suggesting that patients in the SBM group had more aggressive metastatic disease. In our cohort, only two patients (5.0%) had metastases in the hippocampus and peri-hippocampus region, and both were in the SBM group (Table 2 and Figure 1C).

Given the high incidence of BM among NSCLC patients with *ALK* rearrangements, some clinicians have raised the idea of prophylactic cranial irradiation (PCI) for *ALK*-positive patients, even in the absence of BM. In this study, we found that 76.9% of patients in the MBM group had ≤3 BM, none had BM showing diffuse features, and 3 patients had >3 BM (8, 4, 4), of which the lesions were stable or decreasing after chemotherapy or target therapy without local treatment. These findings indicated that more patients in the MBM group were suitable for treatment with SRS, thus avoiding the adverse physiologic and neurologic effects, such as a decline in cognitive function, which commonly occur following WBRT. Therefore, our findings did not advocate the use of PCI for *ALK*-positive NSCLC patients without BM. This approach is further supported by the findings of a recently published study 16 indicating that SRS was an effective salvage strategy for recurrent BM after surgery, even without adjuvant WBRT.

In addition to BM number and size, perilesional edema has been recognized as an important cause of morbidity in patients with brain tumors, including primary and metastatic lesions. A previous study showed that perilesional edema in brain metastases from NSCLC was a predictor of SRS response: minor edema was associated with a better response to SRS treatment and reduced risk of developing new BM, whereas patients with major perilesional edema had a worse response to SRS and an increased risk of out-of-field relapse 17. Another previous study showed that perilesional edema was associated with poor overall survival 18. Thus, in the present study, we analyzed radiological features associated with perilesional edema, including PBES and PBEI. In both groups, patients underwent regular brain MRI follow-up at an interval of 2-3 months after being diagnosed with NSCLC. Further analysis found that patients in the SBM group had more numerous and larger BM with larger PBE, characteristics that reflected SBM harboring aggressive tumor behaviors. These radiological features were consistent with the differences in neurological symptoms observed between the SBM and MBM groups, with only 23.1% of MBM patients initially showing neurological symptoms, which was much lower than in the SBM group (44.4%). These findings highlighted the necessity of routine brain imaging surveillance for *ALK*-positive NSCLC, even without BM.

Hippocampal dysfunction is associated with decreased cognitive function 19. The hippocampal avoidance WBRT (HA-WBRT) technique has been found to decrease the rate of associated side effects in a preliminary analysis 12, and thus has garnered increasing interest in recent years. Given that our findings supported treatment with WBRT for several patients in our study, according to the current guidelines (described above), we further analyzed the feasibility of HA-WBRT in ALK-positive NSCLC patients with BM. Our radiological data showed that the incidence of hippocampal or peri-hippocampal metastases was similar to that of other studies 20, might indicating the feasibility of treatment with HA-WBRT for patients with ALK-positive NSCLC BM. However, more clinical data should be analyzed.

Recently, synergistic effects between radiation and tyrosine kinase inhibitors (TKIs) targeting ALK rearrangement have been demonstrated 21-22 and, under certain circumstances, radiotherapy may improve survival in patients with advanced oncogene-driven NSCLC 23-24. During this study, we screened 187 ALK-positive metastatic NSCLC patients and identified 27 patients with SBM, the synchronous brain metastasis frequency of patients with in ALK-rearranged non-small cell lung cancer is 14.4%. Among these SBM patients, brain radiotherapy prior to crizotinib improved PFS compared with patients who did not receive brain radiotherapy prior to crizotinib 25. This observation supports the hypothesis regarding synergistic effects between radiation and TKIs. Furthermore, despite the availability of several next-generation TKIs, crizotinib remains a first-line treatment option for patients with advanced ALK-positive NSCLC 15 and is widely used in cases where next-generation TKIs are not yet approved or are economically inaccessible. It is therefore imperative to deepen the understanding of radiographic features of BM from ALK-rearranged NSCLC and to determine the optimal timing for incorporating radiation into treatment strategies using various TKIs.

One limitation of the present study was that, despite ALK-targeted TKIs being the standard first-line treatment for advanced NSCLC that is positive for *ALK*-rearrangements, most patients in our study were treated with platinum-based chemotherapy, which may have affected the clinical and radiographic features of patients with MBM. Crizotinib has been approved by the Chinese Food and Drug Administration, but many patients do not have access to this treatment for economic reasons. However, studies have shown that crizotinib has a weak ability to cross the blood-brain barrier, suggesting that first-generation ALK-TKIs might not alter the natural history of NSCLC cells within the central nervous system (CNS) 26. Next-generation ALK-TKIs have better CNS activity and are more effective against BM 27. Nevertheless, not all next-generation TKIs are approved in all countries and are prohibitively expensive for a substantial percentage of patients, with or without medical insurance. Even with next-generation ALK-TKI therapy, BM remains a critical issue for patients with *ALK*-positive NSCLC. More than half of patients with baseline BM (BBM) receiving first-line ceritinib develop initial progression in the brain 28. Among patients with BBM who received alectinib or brigatinib after progressing on crizotinib, the cumulative rates of cranial disease progression were still higher than that of extra-cranial disease progression, commonly exceeding 40% at 24 months 29-30. Updated data from the global phase III ALEX study showed that the median PFS of first-line alectinib was shorter among patients with BBM than those without (27.7 vs. 34.8 months), likely owing in part to the initial progression in CNS 29. Thus, it is necessary to explore the roles of different brain radiotherapy treatment modalities, even in the era of next-generation TKIs, indicating the clinical importance of the current study on the radiographic features of *ALK*-positive NSCLC BM.

## Figures and Tables

**Table 1:** Patient characteristics (N=40)

	ALL	MBM	SBM	*P* value
		N	N	
**Number**	40	13(32.5%)	27(67.5%)	
**Sex**				
Male	22	10(76.9%)	12(44.4%)	0.090
Female	18	3(23.1%)	15(55.6%)	
**Median Age (Years, Range)**	50(22-66)	49(37-66)	52(22-66)	0.909
**PS**				
**0-1**		12(92.3%)	25(92.6%)	1.000
**2**		1(7.7%)	2(7.4%)	
**Smoking history**				
Active and former smoker	5(12.5%)	1(7.7%)	4(14.8%)	0.653
Never smoker	35(87.5%)	12(92.3%)	23(85.2%)	
**Pathology**				
Adenocarcinoma	37(92.5%)	12(92.3%)	25(92.6%)	0.690
Neuroendocrine carcinoma	2(5.0%)	1(7.7%)	1(3.7%)	
Adenosquamous carcinoma	1(2.5%)	0	1(3.7%)	
**Initial Neurological symptoms**				
Yes	15(37.5%)	3(23.1%)	12(44.4%)	0.298
No	25(62.5%)	10(76.9%)	15(55.6%)	
**Treatment before Brain metastasis**		13	0	
Platinum-based Chemotherapy		13(100%)	/	
Crizotinib		3(23.1%)	/	
Others		3(23.1%)	/	
**ALK-rearranged**	40(100%)	13	27	
**EGFR mutation status**				
Wild-type	39(97.5%)	13(100%)	26(96.3%)	
unknown	1(2.5%)	0	1(3.7%)	
**MBR(Observation>6 Months)**	31	8	23	
enlargement	7	1(12.5%)	6(26.1%)	
increase	9	2(25.0%)	7(30.4%)	
>3	8	1	6	
≤3	2	1	1	
stable or decrease	14	5(62.5%)	9(39.1%)	
**Treatment after Brain metastasis**				
Crizotinib without Local treatment		0	6(22.2%)	
WBRT		2(15.4%)	3(11.1%)	
γ knife		2(15.4%)	3(11.1%)	
resection		1(7.7%)	0	

N: Number of Patients; MBM: metachronous brain metastases; SBM: synchronous brain metastases; PS: Performance Status; MBR: modality of brain recurrence; WBRT: whole brain radiotherapy.

**Table 2:** Radiographic analysis (N=40)

	ALL	MBM	SBM	*P*
		N	N	
**Brain metastases**				
≤3	22(55.0%)	10(76.9%)	12(44.4%)	0.090
>3	18(45.0%)	3(23.1%)	15(55.6%)	
**TS(median, mm)**	18.75	12.6	22.3	0.003^*^
**PBES(median, mm)**	41	19	47.5	0.000^*^
**PBEI(Range)**	1.04(0-7.17)	0.24(0-1.75)	1.50(0.269-7.17)	0.001^*^
**Hi+PH metastases**	2(5.0%)	0	2(7.4%)	-

N: Number of Patients; MBM: metachronous brain metastases; SBM: synchronous brain metastases; TS: size of the largest brain tumors; PBES: Peritumoral Brain Edema Sizes; PBEI: Peritumoral Brain Edema Index; Hi+PH metastases: Hippocampus and Peri-Hippocampus region metastases. Asterisk (*) shows a significant difference between MBM and SBM groups (*P*<0.05).
